# Entomopathogenicity to Two Hemipteran Insects Is Common but Variable across Epiphytic *Pseudomonas syringae* Strains

**DOI:** 10.3389/fpls.2017.02149

**Published:** 2017-12-19

**Authors:** Melanie R. Smee, David A. Baltrus, Tory A. Hendry

**Affiliations:** ^1^Department of Microbiology, Cornell University, Ithaca, NY, United States; ^2^School of Plant Sciences, The University of Arizona, Tucson, AZ, United States; ^3^School of Animal and Comparative Biomedical Sciences, The University of Arizona, Tucson, AZ, United States

**Keywords:** *Pseudomonas syringae*, Hemiptera, whiteflies, aphids, pathogen, host–microbe interactions

## Abstract

Strains of the well-studied plant pathogen *Pseudomonas syringae* show large differences in their ability to colonize plants epiphytically and to inflict damage to hosts. Additionally, *P. syringae* can infect some sap-sucking insects and at least one *P. syringae* strain is highly virulent to insects, causing death to most individuals within as few as 4 days and growing to high population densities within insect hosts. The likelihood of agricultural pest insects coming into contact with transient populations of *P. syringae* while feeding on plants is high, yet the ecological implications of these interactions are currently not well understood as virulence has not been tested across a wide range of strains. To investigate virulence differences across strains we exposed the sweet potato whitefly, *Bemisia tabaci*, and the pea aphid, *Acyrthosiphon pisum*, both of which are cosmopolitan agricultural pests, to 12 *P. syringae* strains. We used oral inoculations with bacteria suspended in artificial diet in order to assay virulence while controlling for other variables such as differences in epiphytic growth ability. Generally, patterns of pathogenicity remain consistent across the two species of hemipteran insects, with bacterial strains from phylogroup II, or genomospecies 1, causing the highest rate of mortality with up to 86% of individuals dead after 72 h post infection. The rate of mortality is highly variable across strains, some significantly different from negative control treatments and others showing no discernable difference. Interestingly, one of the most pathogenic strains to both aphids and whiteflies (Cit7) is thought to be non-pathogenic on plants. We also found Cit7 to establish the highest epiphytic population after 48 h on fava beans. Between the nine *P. syringae* strains tested for epiphytic ability there is also much variation, but epiphytic ability was positively correlated with pathogenicity to insects, suggesting that the two traits may be linked and that strains likely to be found on plants may often be entomopathogenic. Our study highlights that there may be a use for epiphytic bacteria in the biological control of insect crop pests. It also suggests that interactions with epiphytic bacteria could be evolutionary and ecological drivers for hemipteran insects.

## Introduction

Most plant-associated bacteria and plant pathogens encounter high environmental heterogeneity and often survive in, and are transmitted through, substrates such as soil, precipitation, bodies of water and leaf litter (Morris et al., 2008, 2013; Monteil et al., 2012, 2013). Some of these species form epiphytic populations in the phyllosphere and may be found in densities averaging 10^5^–10^7^ cells/cm^2^ of leaf surface (Beattie and Lindow, 1995; Andrews and Harris, 2000; Hirano and Upper, 2000; Monier and Lindow, 2004). It is unsurprising then that many phytopathogenic bacteria are phenotypically diverse and have wide habitat ranges. They also utilize diverse host plants with varying destructive phenotypes, such as species of *Dickeya* (soft rot diseases), *Pantoea* (diverse symptoms including leaf spots and rot) and *Pseudomonas* (black speck, soft rot, and others such as leaf spots) (Hirano and Upper, 1990; Buell et al., 2003; Baltrus et al., 2011; Bull et al., 2011). As research on the evolution and interactions of phytopathogens has shifted to include not only pathogenic interactions with plants but phenotypes in diverse habitats, it has become clear that many such bacteria can utilize insect herbivores as vectors (some reviewed in Nadarasah and Stavrinides, 2011) and even as alternative hosts (Grenier et al., 2006; Stavrinides et al., 2009; Nadarasah and Stavrinides, 2011; Hendry et al., 2014). Although the breadth and mechanisms of such entomopathogenic associations are currently poorly understood, it provides a new paradigm that may uncover novel ecological and evolutionary routes for phytopathogenic bacteria.

*Pseudomonas syringae* is a diverse group of strains in the Gammaproteobacteria, and a key model organism for studying epiphytic bacterial growth and molecular mechanisms of virulence to plants (Beattie and Lindow, 1994; Hirano and Upper, 2000; Morris and Kinkel, 2002; Buell et al., 2003; Baltrus et al., 2011, 2012; Lindeberg et al., 2012; McCann et al., 2013; Yu et al., 2013). This is due to high variation in the capabilities of different *P. syringae* strains, which are often phylogenetically very close, but phenotypically distinct (Baltrus et al., 2012). Some closely related strains of *P. syringae* differ remarkably in their virulence to certain host plants and their mechanisms of resistance in the environment (Hwang et al., 2005; McCann et al., 2013). Ecologically, *P. syringae* is thought to utilize various heterogeneous habitats such as bodies of water and precipitation, epiphytic and apoplastic stages, as well as potentially herbivorous insects, and it shows remarkable phenotypic plasticity as it does so (Hirano and Upper, 2000; Monier and Lindow, 2003; Morris et al., 2008; Monteil et al., 2012; Yu et al., 2013). Yet we currently have little understanding of the possible impacts of this environmental heterogeneity on the bacteria, and whether or not trade-offs exist which limit success in varying habitats.

In the last two decades studies have found that in particular, the pea aphid (*Acyrthosiphon pisum*) may be exploited as a host by several species of phytopathogenic and epiphytic bacteria (Harada and Ishikawa, 1997; Grenier et al., 2006; Stavrinides et al., 2009, 2010). Grenier et al. (2006) found that a strain of the phytopathogen *Dickeya dadantii* (formerly *Erwinia chrysanthemi*) was pathogenic to *A. pisum* and contained within its genome homologs of four insecticidal toxins which were thought to play a role in the interaction (Costechareyre et al., 2010, 2012, 2013). Strains of two other members of the *Enterobacteriaceae*, namely *E. aphidicola* and *Pantoea stewartii*, have also been shown to use pea aphids as alternative hosts and grow productively in the gut, and possibly other tissues, to titers approaching 5 × 10^8^ colony forming units (CFU), ultimately leading to host death a few days later (Stavrinides et al., 2010). Recent work by Hendry et al. (2016) demonstrated that the aphid host may alter its reproductive output in response to infection by the bacterium *P. syringae* as a defense mechanism. Using the same two strains of *P. syringae*, Hendry et al. (2014) found they could also infect and multiply within the sweet potato whitefly (*Bemisia tabaci*), despite the effects being ameliorated by a secondary symbiont present in some populations of the insect. Entomopathogenicity has been found previously in related *Pseudomonas* species, although the genes known to be involved are not necessarily also found in *P. syringae* (Vodovar et al., 2006; Ruffner et al., 2012; Kupferschmied et al., 2013). However, the potential prevalence of insect virulence among common phyllosphere strains is somewhat surprising as hemipteran insects are thought to have relatively few interactions with extracellular environmental bacteria (Rosell et al., 2003; Jing et al., 2014). Despite this, previous studies have shown that at least one strain of *P. syringae* (pv. *syringae* B728a) can infect both aphids and whiteflies from epiphytic populations (Stavrinides et al., 2009; Hendry et al., 2014). Yet it remains unclear how widespread and important pathogenicity of plant epiphytes such as *P. syringae* is to hemipteran insect populations.

Prolific crop pests such as aphids and whiteflies (Naranjo et al., 1996; Blackman and Eastop, 2000; Oliveira et al., 2001; Chougule and Bonning, 2012) undoubtedly encounter epiphytic bacteria whilst feeding, and for at least one strain of *P. syringae* we know that ingestion of less than 10 bacterial cells can cause mortality (Hendry et al., 2016). Work to date has demonstrated the impact of only two well-characterized strains of *P. syringae*, namely pv. *syringae* B728a and pv. *tomato* DC3000. The current study uses both these strains, as well as ten more, to investigate possible variation in virulence to two species of Hemiptera: the pea aphid (*A. pisum*) and sweet potato whitefly (*B. tabaci*). Both are highly damaging, cosmopolitan pests of agricultural crops and both have already been shown to be susceptible in some way to strains of *P. syringae*. Infection rates when acquired from bacteria growing epiphytically vary from 1.67 to 5.00% for whiteflies (Hendry et al., 2014) and up to 29% for aphids (Stavrinides et al., 2009). Here we seek to determine firstly whether diverse strains of *P. syringae* vary in their entomopathogenicity, regardless of both the insect species tested and the likelihood of encountering and acquiring infection from epiphytic bacterial populations. Secondly, we investigate whether virulence is consistent across the two species of insect studied. Finally, as a means to elucidate possible trade-offs or linked phenotypes, we characterize the epiphytic growth ability of nine of the strains used.

## Materials and Methods

### Insect Maintenance and Bacterial Cultures

Lab colonies of *A. pisum* were kept at 20°C and a light:dark 16:8 h cycle to represent summer conditions, under which aphids will reproduce parthenogenetically as clones. Colonies were housed in breeding tents containing several fava bean (*Vicia faba*) plants that were rotated out for fresh plants several times a week to keep the population healthy and avoid over-crowding. Clone CWR09/18, collected by Angela Douglas in Freeville, NY, United States in 2009 was used for the current study, and aside from the obligate symbiont *Buchnera aphidicola* it does not harbor any other symbionts (Macdonald et al., 2012). For the experimental assays, aphids were age-controlled on *V. faba* so that all individuals were 5 days old at the start of each assay. To account for possible maternal or host plant effects, aphids were distributed evenly from each of the fava bean plants into the assay dishes.

Whitefly, *B. tabaci*, colonies were kept on cowpea (*Vigna unguiculata*) plants at 27°C after originally being collected by Martha Hunter in Maricopa, AZ, United States in 2006. The invasive Middle East-Asia Minor 1 (MEAM1) lineage was used in this study, as it is established in the field in the United States. All individuals naturally harbored the alphaproteobacterium *Rickettsia* species near *bellii* (*Rickettsia* sp. nr. *bellii*), a widespread secondary symbiont of whiteflies in the United States (Himler et al., 2011). Whiteflies were allowed to multiply to high densities in breeding cages before adults (male and female) were collected and used immediately in experiments. Again, insects were distributed into experimental dishes from across all rearing cages, to account for any environmental effects during rearing time.

All bacterial cultures were grown and kept on King’s B media with added rifampicin (50 ng/μL) as all strains being used displayed resistance to the antibiotic and it ensured that we selected for the correct strains. Culture plates were kept incubated at 27°C and overnight cultures were grown in an incubator shaker set at 27°C and 300 rpm.

### *In Vitro* Pathogenicity Assays

Oral infection assays were used to test the virulence of a total of 12 strains of *P. syringae.* Nine of these strains were tested on both hemipterans (**Table 1**: *P. syringae* pv. *syringae* B728a; pv. *tomato* DC3000; pv. *japonica* MAFF301072 PT; pv. *aptata* DSM50252; pv. *pisi* 1704B; pv. *aceris* M302273; Cit7; TLP2; and *P. savastanoi* pv. *phaseolicola* 1448a), and three more were tested only on whiteflies (**Table 1**: *P. syringae* pv. *maculicola* ES4326; pv. *actinidiae* MAFF302091; and pv. *oryzae* 1_6). These strains represent much of the known phylogenetic breadth of plant pathogenic strains commonly included within the species *P. syringae*, although some have been taxonomically reclassified but are still considered *P. syringae sensu lato* (Sarkar and Guttman, 2004; Bull et al., 2011; Borschinger et al., 2016). Additionally, two strains thought to be non-pathogenic on plants (Cit7 and TLP2) are included. We have focused mainly on strains within phylogroup II, or genomospecies 1 clade (Gardan et al., 1999; Berge et al., 2014) as a strain in this clade was previously found to be highly virulent (Stavrinides et al., 2009; Hendry et al., 2016). All strains were rifampicin resistant isolates provided by David Baltrus and references for origins of strains are found in **Table 1**.

**Table 1:** A summary of *Pseudomonas syringae* strains included in the assays, including epiphytic ability where available alongside levels of pathogenicity to aphids and whiteflies.

Pathovar	Strain	Group	Source	Genbank accession	^∧^Pathogenicity to:		Epiphytic ability
					Whiteflies	Aphids	(CFU/10 leaf disks)
*tomato*	DC3000^∗1^	1	*Solanum lycopersicum*	AE016853	Low	Medium	1.98 × 10^6^
*syringae*	B728a^∗2^	2	*Phaseolus vulgaris*	CP000075	Medium	High	6.87 × 10^6^
*japonica*	MAFF 301072 PT^∗3^	2	*Hordeum vulgare*	AEAH 00000000	Low	Medium	1.35 × 10^6^
*aptata*	DSM50252^∗4^	2	*Beta vulgaris*	AEAN 00000000	Low	Low	1.19 × 10^6^
*aceris*	MAFF 302273 PT^∗5^	2	*Acer* sp.	AEAO 00000000	Low	Low	5.24 × 10^5^
*pisi*	1704B^∗4^	2	*Pisum sativum*	AEAI 00000000	Low	Low	1.49 × 10^6^
N/A	Cit7^∗6^	2	Citrus leaf surface	AEAJ 00000000	High	High	1.32 × 10^7^
N/A	TLP2	2	Potato leaf surface	Gp0012374^♢^	Medium	High	1.45 × 10^6^
*phaseolicola*^‡^	1448a^∗7^	3	*Phaseolus vulgaris*	NC_005773	Low	Medium	1.42 × 10^6^
*actinidiae*	MAFF 302091 PT^∗8^	1	*Actinidia deliciosa*	AEAL 00000000	Low	NA	NA
*maculicola*^¥^	ES4326^∗9^	5	*Raphanus sativus*	AEAK 00000000	Low	NA	NA
*oryzae*	1_6^∗10^	4	*Oryza sativa*	ABZR 00000000	Low	NA	NA

*Group designation and original plant source are given, with GenBank accession numbers for sequences used in phylogenetic tree construction (Borschinger et al., 2016).*

*^∧^Pathogenicity designations taken from significant log rank post hoc pairwise comparisons of survival data. In aphids, there is one overlap between the highest ‘Low’ strain and the lowest ‘Medium’ strain (pv. japonica and pv. aptata were not significantly different). In whiteflies, pv. syringae overlaps with pv. aceris and pv. phaseolicola.*

^‡^*Pathovar is renamed as Pseudomonas savastanoi, not P. syringae.*

^¥^*Strain is renamed as Pseudomonas cannabina pv. asilensis.*

^♢^*JGI project id, not GenBank accession.*

^∗^*References for strain isolation information where available.*

^1^*(Cuppels, 1986)*; ^2^*(Loper and Lindow, 1987)*; ^3^*(Mukoo, 1955)*; ^4^*(Baltrus et al., 2011)*; ^5^*(Sawada et al., 1999)*; ^6^*(Hirano and Upper, 1990)*; ^7^*(Teverson, 1991)*; ^8^*(Takikawa and Inoue, 2003)*; ^9^*(Davis et al., 1991)*; ^10^*(Takeuchi and Munekata, 1992)*.

For each aphid assay, bacterial treatments were prepared by growing an overnight culture that was pelleted, washed in 10 mM MgCl_2_ and then pelleted again and resuspended in 10 mM MgCl_2_ to an OD_600_ of 0.8. Bacterial suspensions were added in a 1:5 ratio to artificial aphid diet (Prosser and Douglas, 1992), with 10 mM MgCl_2_ used for the negative control treatment. This bacterial density has been previously found to result in high rates of infection (Hendry et al., 2016). A 96-well plate was filled with diet, 200 μl per well, and then covered by parafilm to make a feeding sachet. In each well of another 96-well plate, a single age-controlled aphid (5 days old, approximately third instar) was placed and the feeding sachet plate was then inverted above them to allow oral exposure to the bacteria and diet solution, whilst enabling us to record individuals separately throughout the assay. Assay plates were kept at 20°C and after 24 h the feeding sachet was replaced with another sachet of sterile diet only. It should be noted that each strain persisted well in the artificial diet during the 24 h period and there was no indication from dilutions and CFU counts that any of the strains did better or worse than others. The diet was refreshed again after another 24 and 48 h. At these diet changes, and time points between them (0, 6, 24, 30, 48, 55, and 72 h since feeding on sterile diet only) mortality was recorded. An aphid was assumed dead if it had turned brown or was at the bottom of the well (not feeding) and did not move when agitated. Each round of assays included a maximum of six different bacterial treatment plates and a negative control plate, depending on the availability of aphids. Assays were replicated a minimum of three times, using a fresh overnight bacterial culture for each replicate.

Whiteflies were also orally exposed to *P. syringae* strains. For each assay, 5–20 adult whiteflies were placed in 35 mm petri dishes, with a total of 50 to 100 whiteflies per bacterial treatment. Parafilm was stretched over each dish and covered with 1 ml of 15% sucrose in 10 mM sodium phosphate buffer (pH 7.0) and then sealed with another layer of parafilm to create the feeding sachet. Bacterial treatments were made as before, but the final washed pellet was resuspended in 5 ml of sucrose solution. Assays were kept at 24°C for 4–6 days and whitefly mortality was checked twice daily. The same criterion for mortality was applied as for aphids, and a negative control of sucrose in buffer was included in each assay round. Assays were replicated independently at least once, but for most strains twice or more. Whiteflies appeared more robust to external variables such as maternal and host plant effects in comparison with aphids, so although not ideal the number of replications was deemed sufficient to be used in analyses to illustrate patterns alongside the better replicated data on aphids.

### Epiphytic Assays

The rate at which insects encounter epiphytic populations of *P. syringae* is key to its potential effect in the field, and so we quantified the epiphytic ability of nine of the strains tested. Overnight bacterial cultures were prepared as before but resuspended to a final OD_600_ of 1 in approximately 10 ml of 10 mM MgCl_2_. Using a fine mist spray bottle, this was then sprayed until run off onto the upper and lower leaf surfaces of at least six leaf pairs of *V. faba* within a pot of two to three plants. Once fully dried, plants were incubated at 20°C and 90 ± 5% humidity for 48 h. From across the six leaf pairs within each pot of *V. faba* plants, ten 0.5 inch diameter disks were taken using a sterilized cork borer and placed into 10 ml of 10 mM MgCl_2_ buffer, then sonicated for 10 min and vortexed to remove epiphytic bacteria. Serial dilutions were plated and the process was repeated three times for each pot, to allow us to average across replicates to give a CFU count per 10 ml for each strain. The entire process was repeated twice in separate pots of *V. faba* plants for each of the nine strains. Sampling error rate was kept as low as possible by only including CFU counts from dilutions containing 5–50 countable colonies.

### Statistical Analysis

All statistical analysis was conducted in R version 3.3.1 (R Core Team, 2016). Data for each insect species were analyzed separately. Survival analyses were carried out using the R package *survival* (Therneau, 2017) and the assumptions of proportional hazards were visually assessed using plots of the log-log survivor function of the different strains using R package *rms* (Harrell, 2017). Within the Cox Proportional Hazard survival model for each species, bacterial strain was the only variable tested, and rounds of replicates were included as clustering factors to adjust standard errors appropriately. Kaplan–Meier survival curves were drawn to visualize the data (Kaplan and Meier, 1958) and the R package *survminer* (Kassambara and Kosinski, 2017) was used to conduct log rank tests of pairwise comparisons between strains, using the Bonferroni method of correcting for multiple tests. These log rank tests make no assumptions about the survival distributions and were used to categorize strains into ‘low,’ ‘medium,’ or ‘high’ pathogenicity level.

Data on epiphytic ability were log_10_-transformed for tests of Pearson’s correlation coefficient with virulence data. Transformed epiphytic data were also used in ANOVA analysis, with Tukey’s HSD *post hoc* test to determine if strains were differentially capable of colonizing plant leaf surfaces. The non-parametric Spearman’s rank correlation was used to test for a relationship between pathogenicity across the two insect species.

## Results

### Pathogenicity Assays

**FIGURE 1:**
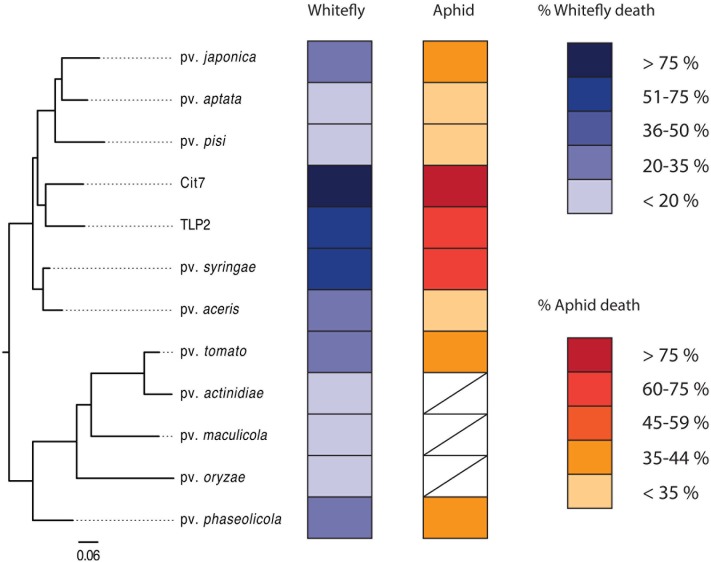
A phylogenetic tree showing all 12 strains used in the study, alongside their virulence to both whiteflies and aphids. White boxes denote where data is not available for those strains. Separate keys indicate virulence to each insect as percentage dead at 72 h post-infection. The phylogenetic tree was constructed in PhyloPhlAn (Segata et al., 2013) using 399 of the most conserved protein sequences across the genomes of the 12 strains, encompassing 1969 informative sites. Protein sequences were obtained from GenBank (see **Table 1**).

**FIGURE 2:**
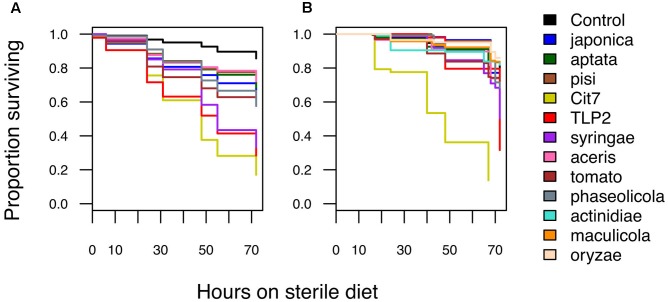
Kaplan–Meier survival graphs showing the proportion of surviving individuals over time for **(A)** aphids and **(B)** whiteflies after oral exposure to different *Pseudomonas syringae* strains. Hours post-infection started when individuals were moved onto fresh sterile diet for the first time. All data taken until 72 h is shown here and used in further analyses.

Pathogenicity of *P. syringae* strains varied widely across both hemipteran species. Average death at 72 h after exposure ranged from 14 to 86% in *B. tabaci* and from 26 to 83% in *A. pisum* (**Figure 1**). The pathogenicity of strains was highly correlated between whiteflies and aphids, with both insects being more susceptible to strains Cit7, TLP2 and pv. *syringae* B728a and least susceptible to pv. *aptata* DSM50252 and pv. *pisi* 1704B (Spearman’s rank correlation: *S* = 8.07, *p* < 0.001, *r* = 0.93). Although we see a tight correlation between mortality at 72 h within the two species of Hemiptera studied, this does not necessarily mean the absolute values are the same across species, but that the relative death caused by different strains of *P. syringae* shows the same pattern. To illustrate this, the pathogenicity of strains was grouped into ‘low,’ ‘medium,’ and ‘high’ categories specific to each insect species using log rank *post hoc* comparisons of survival curves (**Table 1**). The general rate of death also seems to differ somewhat between aphids and whiteflies. Although Cit7 seems to be fast-acting in both species and shows a continual high daily mortality, other strains such as TLP2 and pv. *syringae* B728a show an early and continual decline in aphids, but a delayed effect in whiteflies, despite coming to similar end points (**Figure 2**). As the Cox Proportional Hazards analysis is quite conservative, and possibly due to this slower mortality rate in whiteflies, only the two non-plant pathogens showed a significantly different survival rate to the negative control within whiteflies (**Table 2**). Contrary to this, aphid survival rates were significantly different for almost all strains when compared to the negative control, with only pv. *pisi* having no discernable effect on the rate of mortality (**Table 2**).

**Table 2:** Survival ranges for both insect species infected with different strains of *P. syringae*.

Pathovar	Strain	Aphids			Whiteflies		
		*Z*_df__=__9_	HR	Survival range^¥^	*Z*_df__=__12_	HR	Survival range^¥^
Control	–	–	–	0.84–0.87	–	–	0.72–0.81
*tomato*	DC3000	7.71	3.76^∗∗∗^	0.53–0.63	0.36	1.23	0.68–0.80
*syringae*	B728a	9.80	6.58^∗∗∗^	0.28–0.39	1.62	2.32	0.44–0.57
*japonica*	MAFF 301072 PT	5.52	3.19^∗∗∗^	0.58–0.65	-0.15	0.92	0.70–0.85
*aptata*	DSM50252	5.00	2.50^∗∗∗^	0.65–0.72	0.63	1.19	0.67–0.82
*aceris*	MAFF 302273 PT	2.49	2.17^∗^	0.69–0.76	0.19	1.11	0.65–0.84
*pisi*	1704B	1.55	2.02	0.70–0.79	-0.36	0.82	0.72–0.88
N/A	Cit7	16.28	10.31^∗∗∗^	0.13–0.22	3.74	13.06^∗∗∗^	0.07–0.26
N/A	TLP2	9.06	7.99^∗∗∗^	0.24–0.34	2.03	3.16^∗^	0.24–0.42
*phaseolicola*	1448a	5.12	3.52^∗∗∗^	0.53–0.62	0.54	1.35	0.61–0.77
*actinidiae*	MAFF 302091 PT	NA	NA	NA	-0.43	0.75	0.75–0.90
*maculicola*	ES4326	NA	NA	NA	-0.65	0.65	0.78–0.90
*oryzae*	1_6	NA	NA	NA	-1.06	0.55	0.81–0.92

*Statistics are based on Cox proportional hazard analysis, and the hazard ratio is included to indicate effect size (HR: 1 = no effect; <1 = a reduction in the hazard; >1 = increase in the hazard).*

^¥^*95% confidence intervals for survival probability at 72 h post exposure.*

^∗^*Significantly decreased survival compared to the control at p < 0.05.*

^∗∗∗^*Significantly decreased survival compared to the control at p < 0.001.*

### Epiphytic Assays

**FIGURE 3:**
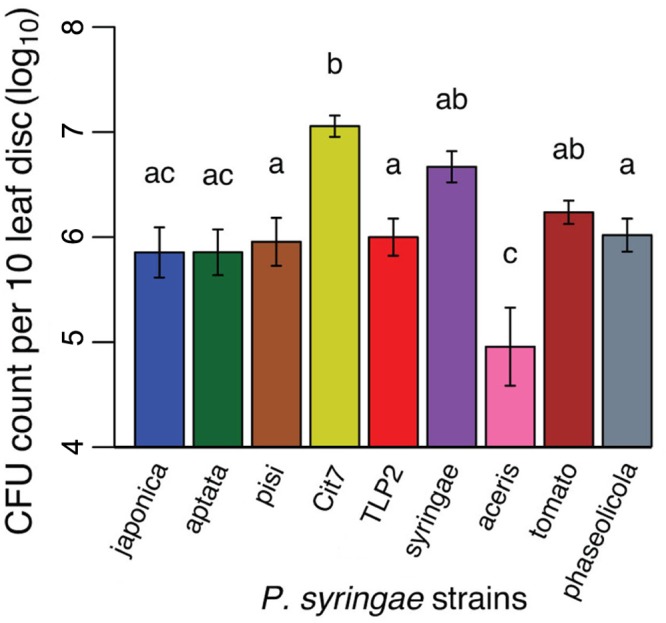
Epiphytic ability of nine of the *P. syringae* strains used in the virulence assays, measured as CFU counts per 10 leaf disks sampled. Given our accuracy cut offs of 5–50 colony counts in the methodology, the *y*-axis starts at the minimum CFU count we might see in our dilutions. The absolute minimum of 5 CFUs in an undiluted 5 μl droplet would equate to 10,000 CFUs in the 10 ml sample taken.

All nine strains tested demonstrated an ability to epiphytically colonize fava bean leaves, and persist for at least 48 h. There was a substantial amount of variation in the epiphytic ability of the strains tested, with CFU counts per ten leaf disks sampled averaging from 5.24 × 10^5^ (pv. *aceris*) to 1.32 × 10^7^ (Cit7; **Figure 3** and **Table 1**). Across all strains tested, there was a significant difference in their epiphytic ability (ANOVA; *F*_8,47_ = 8.02, *p* < 0.001). Tukey’s HSD *post hoc* tests showed that at a significance level of 0.05, most strains persisted within a similar range, apart from pv. *aceris* which differed from all others except pv. *japonica* and pv. *aptata* at the lower level, and Cit7 which differed from all but another non-plant pathogen TLP2 and the highly phytopathogenic pv. *syringae* B728a, at the uppermost epiphytic level (**Figure 3**). We note that these assays included high founding population densities and were kept at high humidity, so these values may represent the maximum epiphytic ability of strains on fava plants; typical epiphytic ability in nature may be lower.

**FIGURE 4:**
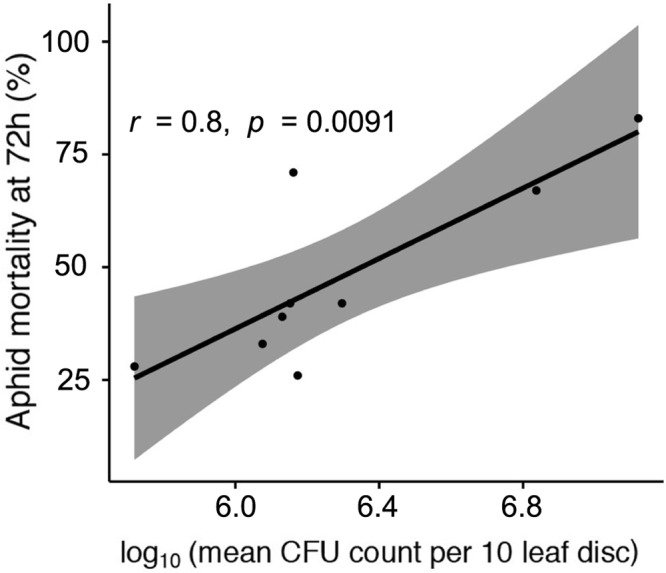
Correlation between aphid mortality at 72 h post-infection, and log_10_-transformed epiphytic ability of strains measured as mean CFU count per 10 leaf disks taken. Gray area denotes 95% confidence intervals.

It is also clear that the better the strain is at establishing epiphytic colonies the more virulent it is to pea aphids 72 h after initial infection (**Figure 4**, Pearson’s product-moment correlation: *t* = 3.57, d.f. = 7, *p* < 0.01, *r* = 0.80). This does not demonstrate cause and effect, but that perhaps that there is a common trait important for success, or proliferation, of the bacteria in both environments.

## Discussion

*Pseudomonas syringae* is known to be a very successful and ecologically adaptable group of bacteria, and here we have illustrated that although little is known about their use of insects as potential reservoirs it could be a very important association for both partners. Across 12 pathovars of *P. syringae*, spanning much of the genomic diversity available, we find that pathogenicity toward both aphids and whiteflies varies substantially from having no significant effect when compared to negative controls, to killing over 80% of individuals within 4 days. We also found variation in the epiphytic ability of different pathovars, which interestingly seems to correlate with their pathogenicity toward at least the two species of insect tested here. Until recently it was thought that aphids at least could pick up infections from plants with high epiphytic populations of *P. syringae* (Stavrinides et al., 2009), but for the well-studied and highly virulent strain *P. syringae* pv. *syringae* B728a it has been estimated that the minimum infective dose could be less than 10 cells (Hendry et al., 2016), similar to that estimated for the phytopathogen *D. dadantii* (Grenier et al., 2006). If such is the case for all strains of *P. syringae* and other phytopathogenic bacteria, then the interaction between even low-level epiphytic populations and herbivorous insects could be a crucial ecological and evolutionary driver of both host and pathogen.

Epiphytic success is integral to the life cycle of many *P. syringae* pathovars. The vast majority of plants worldwide serve as hosts to this common pathogen (Hirano and Upper, 2000; Baltrus et al., 2014, 2017). The majority of work to date has focused on the question of what determines whether, and how successfully, a pathogen infects plant tissue (Baltrus et al., 2011, 2012; Lindeberg et al., 2012; McCann et al., 2013). More recently has this focus begun to include the mechanisms associated with epiphytic populations of phytopathogens as well (Beattie and Lindow, 1994; Morris and Kinkel, 2002; Yu et al., 2013). Epiphytic populations are known to be correlated with and act as reservoirs for disease in plants (Hirano and Upper, 2000; Monteil et al., 2013). Fernández-Sanz et al. (2016) showed that populations, transient as they may be, on neighboring non-host plant communities may act as a reservoir for invading strains of *P. syringae.* This suggests that although *P. syringae* is widespread and disperses long distances in precipitation, local dispersal between neighboring plants may also have important implications. It may be that insects can act as potential reservoirs for the bacterium, as they are able to pick up cells through feeding and spread bacteria to neighboring plant communities (Stavrinides et al., 2009). For aphids in particular, the excretion of honeydew has been shown to deposit high densities of bacteria onto leaves, averaging 2 × 10^7^ CFU per aphid after feeding on epiphytic populations of *P. syringae* (Stavrinides et al., 2009), and so may provide efficient small-scale transmission of the phytopathogen amongst other insect and plant hosts.

However, if the strain was highly proficient at establishing epiphytic populations and dispersal via a multitude of routes, such as *P. syringae* strains often are (Lindow and Brandl, 2003; Morris et al., 2008; Monteil et al., 2012), then it is not necessarily of huge importance to use possible insect hosts. Becoming ‘trapped’ inside an insect host could be lowering dispersal potential if other routes are more efficient, and so there would be selection on highly epiphytic strains to kill any insect host as quickly as possible. One of the most virulent strains to insects, which form the highest density of epiphytic colonies as well, is also non-pathogenic to the plant host from which it was isolated (Cit7). Strain Cit7 is found naturally occurring on leaf surfaces and it is very possible that whatever makes it good epiphytically, may well influence its ability to colonize the gut of hemipterans too. There is no evidence to date to suggest that the strong correlation we show here between the virulence of a strain and its epiphytic ability (**Figure 4**) is anything other than coincidence. It is indeed possible that despite their ability to grow inside insect hosts, it may well be an ecological and evolutionary dead end for strains of *P. syringae*.

The two species of Hemiptera studied here share many life history similarities, such as being sap-sucking insects with high reproductive rates, and a large propensity for dispersal. Even with missing data for some of the 12 strains in aphids, the patterns of pathogenicity seen are highly consistent (**Figures 1**, **2**). Another commonality is that the bacterial diversity and abundance of gut communities in both insects is extremely low, ranging from 3 to 7 operational taxonomic units (OTUs) found after pyrosequencing 16S rRNA amplicons (Jing et al., 2014). It is possible that if phytopathogens such as *P. syringae* are able to initially colonize the gut of these insects then they may be able to take advantage of the resources available and lack of competitive exclusions, and hence multiply rapidly and successfully without being halted by the host. Pea aphids are somewhat able to elicit an immune response to bacteria (Laughton et al., 2011; Costechareyre et al., 2013), but show a reduced immune response in comparison to some other insect species due to lacking a number of known immune pathways (Gerardo et al., 2010; International Aphid Genomics Consortium, 2010; Altincicek et al., 2011). Plant viruses transmitted by whiteflies have been shown to activate certain antimicrobial peptides within their host (Luan et al., 2011), and orally toxic *Photorhabdus* bacterial species also appear to up-regulate certain genes associated with immunity within adult whiteflies (Shrestha and Lee, 2012), although these studies are limited. It is possible that immunological defenses within aphids especially are generally lacking and may help explain the patterns seen here, but whether or not this reasoning extends across other hemipterans is difficult to say currently.

The patterns of virulence seen here may well be shared across hemipterans, particularly if there is a tendency to invest in strategies such as reproduction as a trade-off to lacking immune defenses (Leventhal et al., 2014; Hendry et al., 2016). The ubiquity of phytopathogens in the phyllosphere, and the emerging confirmation that insects may be used as alternative hosts or short-term reservoirs, would suggest that these susceptible species should evolve some response in defense. It is likely that such a response could be non-immunological, such as a behavioral adaptation, or otherwise aided by other resident species of symbiotic bacteria (Parker et al., 2011). It is necessary to note that the *B. tabaci* individuals used here were all positively infected with the secondary symbiont *Rickettsia*. The relative levels of mortality across the strains seen here should not be affected, but the overall rate may be lower than if *Rickettsia* was not present (Hendry et al., 2014).

The biological control of agricultural pests is a rapidly evolving field and new solutions are sought constantly. Entomopathogenic bacteria such as *Bacillus thuringiensis* and *Photorhabdus*/*Xenorhabdus* species are highly virulent to insect pests, but have limited use as they cannot persist unaided in the environment (Kupferschmied et al., 2013). To determine the true potential of *P. syringae* strains in biocontrol strategies, and the likelihood of a fully predictive framework, it is essential that more potential hosts and mechanisms be investigated. Evidence of *P. syringae* virulence can be seen in other aphid species and to a lesser extent, and through different routes, in other insect groups (Hendry, unpublished). If such a wide-ranging phytopathogen can be infective at minimal epiphytic densities, reduce herbivore populations and in some instances cause no detrimental effects to host plants, then there are clearly worthwhile routes of investigation to take in the future.

## Author Contributions

TH and DB conceived the idea and contributed to experimental design. MS contributed to experimental design and carried out aphid experimental work, overall data analysis, and writing of the manuscript. TH carried out whitefly experimental work and contributed to interpretation of results and writing of the manuscript. All authors read and approved the final draft.

## Conflict of Interest Statement

The authors declare that the research was conducted in the absence of any commercial or financial relationships that could be construed as a potential conflict of interest.
